# Mechanisms of Self-Assembly of Giant Unilamellar Vesicles in the Army Liposome Formulation (ALF) Family of Vaccine Adjuvants

**DOI:** 10.3390/pharmaceutics17091092

**Published:** 2025-08-22

**Authors:** Calin Nicolescu, Essie Komla, Mangala Rao, Gary R. Matyas, Carl R. Alving

**Affiliations:** 1 U.S. Military HIV Research Program, Center for Infectious Disease Research, Walter Reed Army Institute of Research, 503 Robert Grant Avenue, Silver Spring, MD 20910, USA; ekomla@hivresearch.org (E.K.); mrao@hivresearch.org (M.R.); gmatyas@hivresearch.org (G.R.M.); 2 Henry M. Jackson Foundation for the Advancement of Military Medicine, 6720A Rockledge Drive, Bethesda, MD 20817, USA

**Keywords:** liposome, membrane fusion, giant unilamellar vesicle, lyophilization, monophosphoryl lipid A, nonlamellar lipid, ALFQ, adjuvant, QS21

## Abstract

**Background/Objectives:** Army Liposome Formulation with QS21 (ALFQ) is a vaccine adjuvant formulation consisting of liposomes that contain saturated zwitterionic and anionic phospholipids, 55 mol% cholesterol, and small molar amounts of monophosphoryl lipid A (MPLA) and QS21 saponin as adjuvants. A unique aspect of ALFQ is that after addition of QS21 to nanoliposomes (<100 nm), the liposomes self-assemble through fusion to form giant (≥1000 nm) unilamellar vesicles (GUVs). The purpose of this study was to introduce and investigate new intermediate structures in the fusion process that we term tethered incomplete microspheres (TIMs), which were discovered by us incidentally as structures that were visible by phase contrast microscopy. **Methods:** Differential centrifugation; phase contrast microscopy; confocal microscopy of vesicles or TIMs which contain fluorescent chromophores linked to phospholipids or cholesterol; ultra-performance liquid chromatography-tandem mass spectrometry (UPLC-MS/MS) analysis of lipid components of liposomes and TIMs; and dynamic light scattering were all used for the characterization of TIMS. **Results and Conclusions:** (A) Sizes of TIMs range from overall aggregated structural sizes of ~1 µm to mega sizes of ≥200 µm. (B) Stable TIM structures occur when a fusion process is stopped by depletion of a fusogenic lipid during an evolving fusing of a lipid bilayer membrane. (C) TIMs consist of long-term stable (>2 years), but also metastable, tightly aggregated tear-drop or spherical incomplete GUVs tethered to visible masses of underlying vesicles that are not individually visible. (D) The TIMs and GUVs all contain phospholipid and cholesterol (when present) as bulk lipids. (E) Lyophilized liposomes lacking QS21 saponin, but which still contain MPLA (ALF55lyo), also self-assemble to form GUVs and TIMs. (F) Cholesterol is a required component in nanoliposomes for generation of GUVs and TIMs by addition of QS21. (G) Cholesterol is not required for production of GUVs and TIMs in ALFlyo, but cholesterol greatly reduces and narrows the polydisperse vesicle distribution.

## 1. Introduction

Army Liposome Formulation with QS21 (ALFQ) is a potent and safe vaccine adjuvant that contains monophosphoryl lipid A (MPLA) and QS21 saponin, which serve as immunostimulants to enhance innate immunity [1]. The initial purpose of this study was to elaborate on our previous work, which explored the mechanisms of transfer of QS21 saponin, cholesterol, and MPLA in the self-assembly of ALFQ and the discovery of giant unilamellar liposomes (GUVs) in ALFQ [2]. Unexpectantly, in this follow-on study, we have discovered here the presence of mega-size groups of previously unrecognized stable intermediate phospholipid membrane structures in ALFQ; we refer to the mega-size structure as a tethered incomplete microsphere (TIM). The discovery here of TIMs as intermediate structures provides insight into the liposomal fusion process leading to GUVs in ALFQ. The discovery of TIMs could also provide understanding of underlying mechanisms of liposomal nonlamellar lipids, in general, as vaccine adjuvants for stimulation of innate immunity.

In 1989, in response to widespread use of microbial substances, such as bacterial lipopolysaccharide and other microbial materials as vaccine adjuvants, providing huge insight into innate immunity, the immunologist Charles Janeway argued that adjuvants [containing microbial substances] are “the immunologist’s dirty little secret” [3]. This led Janeway to propose the existence of pathogen-associated molecular patterns (PAMPs) and pattern recognition receptors (PRRs) as structures that activate the innate immune systems. This, together with the discovery of dendritic cells as antigen presentation cells by Steinman [4], generated considerable scientific and commercial interest, and both are considered major advancements in the fields of innate immunity and vaccine adjuvant mechanisms.

In 2011, the importance of the mechanisms of lipid A interactions with cells was emphasized when Bruce Beutler and Jules Hoffmann shared a Nobel Prize for the discovery of Toll-like receptor 4 (TLR4), a cellular membrane receptor for bacterial lipopolysaccharide with lipid A as its TLR4 binding site, and Ralph Steinman also received a Nobel Prize for the discovery of dendritic cells. This led to the recognition of numerous other types of cellular receptors, such as damage- or danger-associated molecular patterns (DAMPs), as well as other receptors and structures, leading to the activation of innate immunity [5,6,7]. Purified or synthetic lipid A and MPLA have long histories as stimulators of innate immunity and as vaccine adjuvants [8,9]; the first phase 1 human trial of a vaccine with liposomes containing MPLA as an adjuvant was described in 1992 [10].

In 1962, Bangham and associates introduced plant saponins as potential constituents in liposomes [11], and immunomodulatory effects of various types of saponins have been discovered [12]. Liposomes containing QS21 saponin derived from *Quillaja saponaria* soapbark trees, together with MPLA, are now present as constituents in a GSK liposomal adjuvant grouping known as adjuvant system AS01. Development of AS01 has led to three AS01-containing adjuvanted vaccines licensed by the U.S. Food and Drug Administration and other regulatory agencies [6,7,13].

It is against this background that Army Liposome Formulation with QS21 saponin (ALFQ) was developed [14,15]. ALFQ is distinctly different than AS01 in that the bulk phospholipid bilayer of ALFQ contains two saturated phospholipids, one of which is neutral while the other is anionic; ALFQ contains 55 mol% cholesterol when compared to the total bulk phospholipids. However, another unique characteristic of ALFQ is that it has a polydisperse size distribution, with vesicle diameters ranging from ~50 nm to ~30,000 nm. ALFQ liposomes with diameters ≤ ~100 nm are referred to as small unilamellar vesicles (SUVs); those with diameters ≥ 1000 nm are referred to as giant unilamellar vesicles (GUVs). In contrast to ALFQ, the bulk liposomal phospholipids of the AS01 adjuvant system comprise only unsaturated and uncharged phospholipids; AS01 has 33.4 mol% cholesterol compared to the total phospholipids, and it has SUVs with a uniform diameter size of ~100 nm [16]. ALFQ has exhibited potency and safety in two phase 1 studies as a constituent of a malaria vaccine [17] and a phase 1 SARS-CoV-2 vaccine [18]; nine other phase 1 studies are also in preparation or in progress.

To examine the structure of ALFQ in more detail, a previous mass spectroscopy analysis of differentially centrifuged polydisperse ALFQ vesicles revealed that GUVs and SUVs each contained similar molecular amounts of phospholipids, but the GUVs contained ≥ 90% of the total amounts of QS21 saponin, cholesterol, and MPLA, and the SUVs contained ≤ 10% of the total QS21, cholesterol, and MPLA [2].

In addition to ALFQ, lyophilized SUVs that contained MPLA in addition to dimyristoyl phosphatidylcholine (DMPC), dimyristoyl phosphatidylglycerol (DMPG), and 43% cholesterol (ALF43), but lacked QS21 saponin, upon rehydration (ALFlyo) contained GUVs [19]. Here we found also that ALF55lyo (containing 55% cholesterol) had both GUVs and stable TIMs, with the TIMs as a presumed intermediate stage in the generation of the GUVs. We conclude that, in the self-assembly of liposomes containing phospholipids and cholesterol, with a small molar fraction of nonlamellar lipids, the presence of TIM structures is a frequent intermediate stage, and a stable byproduct as the liposomes transition in size from SUVs to GUVs.

## 2. Materials and Methods

### 2.1. Materials and Reagents

DMPC, DMPG, synthetic MPLA (3D-PHAD^®^), and cholesterol (plant-derived), along with fluorescently labeled (TopFluor^™^) cholesterol, 18:1 cyanine 5.5 phophoethanolamine, and 18:1 carboxyfluorescein phosphoethanolamine, were purchased from Avanti Research (Croda), Alabaster, AL, USA. Fluorescently labeled Texas Red^™^ PE and Oregon Green^™^ 488 PE were purchased from Thermo Fisher Scientific, Eugene, OR, USA. QS21 was purchased from Desert King International, Chula Vista, CA, USA. DMPC, cholesterol, and fluorescent lipids were dissolved in freshly distilled chloroform, while DMPG and MPLA were dissolved in chloroform:methanol (9:1). Methanol, water, isopropanol (IPA), dichloromethane, formic acid, and ammonium formate (all Optima^TM^ LC-MS grade) were purchased from Fisher Scientific, Fair Lawn, NJ, USA.

### 2.2. ALF55 and ALFQ Formulation Methods

**Figure 1:**
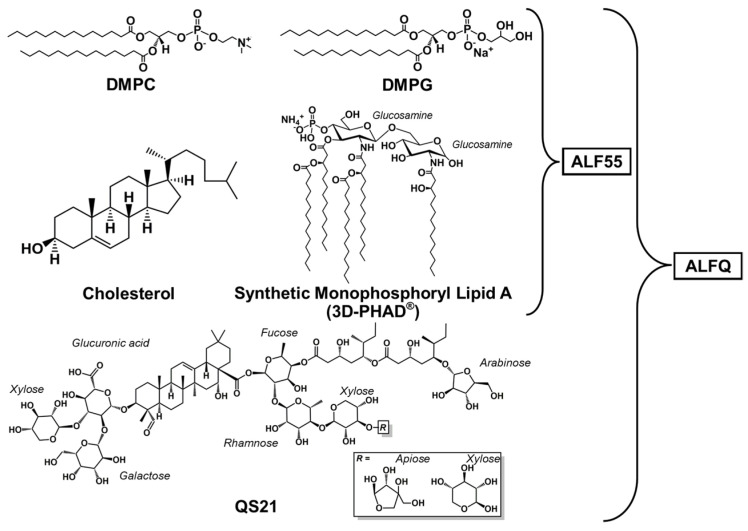
Lipid constituents of Army Liposome Formulation (ALF) containing 55 mol% cholesterol (ALF55) and ALF55 with QS21 (ALFQ).

ALF55 and ALFQ liposomes were formulated with lipid constituents (Figure 1) using previously described methods [2]. In short, a lipid film containing DMPC, DMPG, 55 mol% cholesterol relative to the phospholipids (DMPC + DMPG), and MPLA was dried and rehydrated in Sorensen’s Phosphate-Buffered Saline (SPBS) with pH 6.2. This liposome formulation is ALF55, and it was microfluidized to obtain SUVs. Then, ALF55 liposomes were incubated with QS21 to obtain ALFQ. The molar ratios of DMPC:DMPG:Chol:MPLA:QS21 in the liposomes were 9:1:12.2:0.114:0.044. ALF liposomes were prepared the same way, but cholesterol was omitted from the lipid mixture (ALF0). To create liposomes with fluorescent lipids, 0.25 mol% (relative to DMPC + DMPG) of each labeled phospholipid was incorporated into the lipid mix at the start of their respective formulations to obtain either ALF55 or ALF0 labeled with fluorescent lipids.

### 2.3. Lyophilized ALF55 Formulation Methods

**Figure 2:**
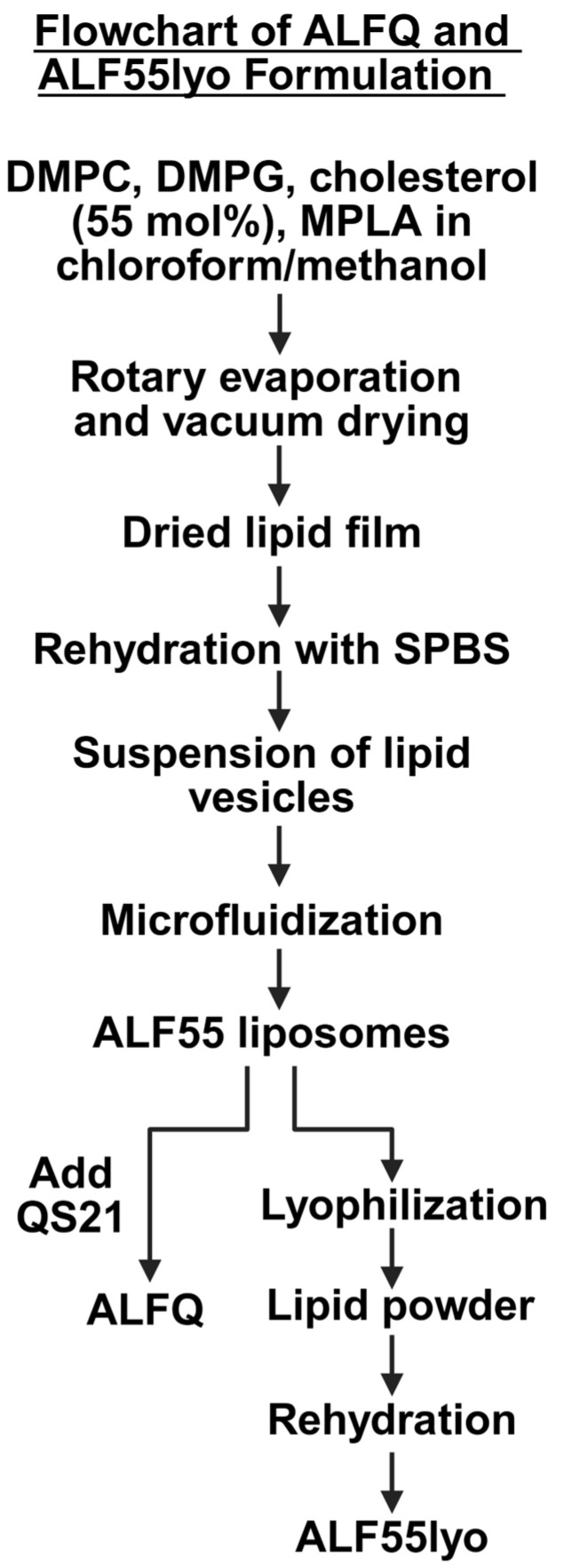
A flowchart depicting the process for ALFQ and ALF55lyo formulation. Both preparations follow the same initial steps to reach ALF55 liposomes, after which they diverge when QS21 is added to obtain ALFQ, or when they are lyophilized and rehydrated to obtain ALF55lyo.

ALF55 liposomes were prepared as in Section 2.2 and Figure 2 and aliquoted into 250 µL volumes for lyophilization on a VirTis Advantage EL-85 freeze dryer (ATS Scientific Products, Warminster, PA, USA). Samples were first frozen at –45 °C for 90 min, then underwent a primary drying step under 100 mbar vacuum at a temperature gradient from –40 °C to +10 °C, and a secondary drying step at +10 °C. The resulting powder was rehydrated in its original volume of SPBS.

### 2.4. Microscopy

Phase contrast images of the ALFQ TIM formation were acquired using an Olympus BH2 microscope with a 40X/0.65 DPlan objective and an Olympus DP71 camera (Evident Scientific, Waltham, MA, USA). Confocal fluorescence microscopy was performed with an Olympus Fluoview FV1200 laser scanning confocal microscope with a 40X/1.30 Oil UPlanFL N and 60X/1.42 Oil PlanApo objective (using the latter where specified). Images were processed using ImageJ software (Version 1.54p) by adjusting window and level settings to better visualize image contrast.

### 2.5. Isolation of SUVs from GUVs + TIMs in ALF55lyo

An ALF55lyo formulation at a concentration of 22.9 mM phospholipid was diluted 1:10 in SPBS to reach a total volume of 5 mL. Samples were transferred to 15 mL glass tubes and centrifuged in a Sorvall RC5C centrifuge (Thermo Scientific, Waltham, MA, USA) at 8000 rpm (767× *g*) for 10 min at 22 °C. The supernatant containing SUVs was collected, and the pellet containing TIMs + GUVs was washed three times with SPBS. Both fractions were analyzed by ultra-performance liquid chromatography–high-resolution tandem mass spectrometry (UPLC-MS/MS) to determine their compositions of phospholipids, cholesterol, and MPLA.

### 2.6. UPLC-MS/MS Analysis of Liposome Components

Liposomal lipid components were measured with a UPLC-MS/MS-based method on a Thermo Scientific Vanquish UPLC coupled with a Q-Exactive Quadrupole-Orbitrap detector as previously described [2,20,21]. The phospholipids DMPC and DMPG, along with MPLA, were analyzed using similar methods. An Agilent Zorbax Eclipse Plus C18 column (4.6 mm ID × 50 mm, 1.8 μm particle size) was used for separation in both cases, with the injection volume set to 1 µL. Mobile phases of 95/5 methanol/water (A) and IPA (B), with 5 mM ammonium formate and 0.1% formic acid, were used at a constant flow rate of 0.5 mL/min and a column temperature of 45 °C. For cholesterol quantification, separation was performed in a Kinetex^®^ Phenomenex C18 column (2.1 mm ID × 150 mm, 2.6 µm particle size), using mobile phases of 95/5 methanol/water (A) and 62/36/2 methanol/dichloromethane/water (B), with 5 mM ammonium formate and 0.1% formic acid, at a constant flow rate of 0.4 mL/min and a column temperature of 40 °C.

The electrospray and source settings were as follows: 2.5 kV (capillary voltage), 320 °C (capillary temperature), 25 AU (sheath gas flow rate), 10 AU (Aux gas flow rate), and 300 °C (Aux gas temperature). Data were acquired using negative electrospray ionization in parallel reaction monitoring (PRM) mode. DMPC and DMPG were detected using PRM transitions of *m*/*z* 772.50 > 227.20 at 5.44 min and 665.44 > 227.20 at 3.85 min, respectively. Quantification was performed using an external calibration method with a 1/x weighting scheme in TraceFinder 5.1 (Thermo Fisher Scientific). MPLA was detected using a PRM transition of *m*/*z* 1518.08 > 1004.65 at 13.07 min. Quantification was performed using an external calibration method with an equal weighing scheme. Cholesterol was detected using a PRM transition of *m*/*z* 369.35 > 147.12 at 4.26 min. Quantification was performed using an external calibration method with an equal weighing scheme in TraceFinder 5.1.

### 2.7. Dynamic Light Scattering

The hydrodynamic diameter size distribution of liposomes was measured using a Malvern Zetasizer Nano S (Malvern Panalytical, Westborough, MA, USA). Liposome formulations were diluted 1:50 in SPBS and equilibrated to 25 °C before measurement. Results were presented as an average of three measurements and displayed by intensity distribution. Zeta potentials were similarly measured in triplicate at the same dilution.

## 3. Results

### 3.1. Tethered Incomplete Microspheres (TIMs) as a Presumed Intermediate Step and as a Byproduct in the Formation of GUVs

#### 3.1.1. Initial Fusion Process Leading to Free-Floating GUVs in ALFQ

As detected by phase contrast microscopy, after addition of QS21 to a clear suspension of ALF55 SUVs, initial cloudiness occurred within seconds (Video S1). This cloudiness was initially due to sudden emergence of large, agglomerated masses of vesicles in which the individual nanovesicles were not yet visible. In the cloudy background, large numbers of GUVs became visible. However, also detected unexpectantly were unique mega-size structures of various sizes, some > 200 µm, which consisted of aggregations of numerous giant unilamellar round and tear-drop structures that were attached to underlying dense ill-defined central cores (Video S1). This new type of mega-size structure is referred to here as a TIM.

**Figure 3:**
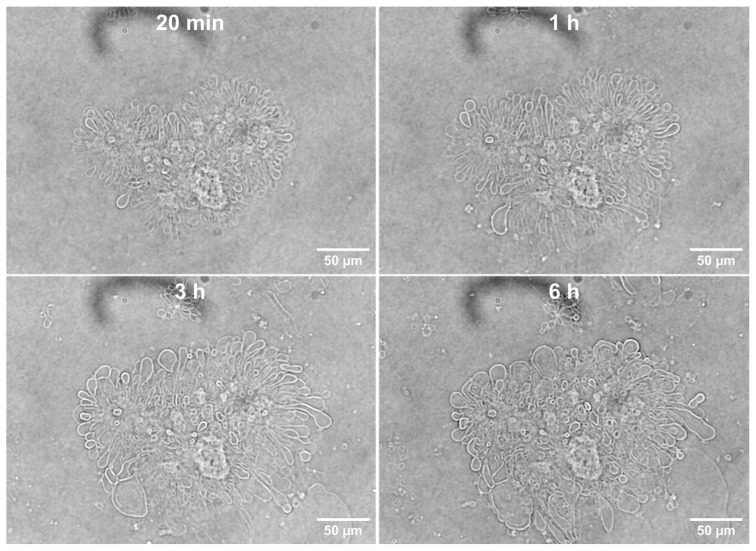
Appearance of tethered incomplete microspheres (TIMs) in ALFQ at different times after completion of the fusion reactions as visualized by phase contrast microscopy.

The complete fusion and budding reactions that resulted both in the free-floating GUVs and TIMs were essentially completely finished within ≤20 min (Figure 3). After the fusion event caused by the addition of QS21 saponin to unilamellar ALF55 vesicles was completed, all of the GUVs and TIMs that remained were stable, and the TIMs showed no evidence of further budding into free-floating GUVs, even after 6 h or more (Figure 3). From the timing of the fusion process, we conclude that the TIM structures that became visible by phase contrast microscopy had been permanently halted in the process of budding into independent free-floating vesicles.

#### 3.1.2. Detection of Phospholipids and Cholesterol in ALFQ TIMs

In order to visualize the chemical components of TIMs in ALFQ, two different phospholipid-bound chromophores were employed, one green (Oregon Green™ DHPE) and the other red (Texas Red™ DHPE). Each chromophore was linked to the phosphate moiety of phospholipid, and each was incorporated independently into two separate groups of ALF55 nanoliposomes. The two labeled ALF55 nanoliposome groups were then mixed together and exposed to QS21 saponin to initiate fusion. Using two different phospholipid-linked chromophore colors increased visual clarity.

**Figure 4:**
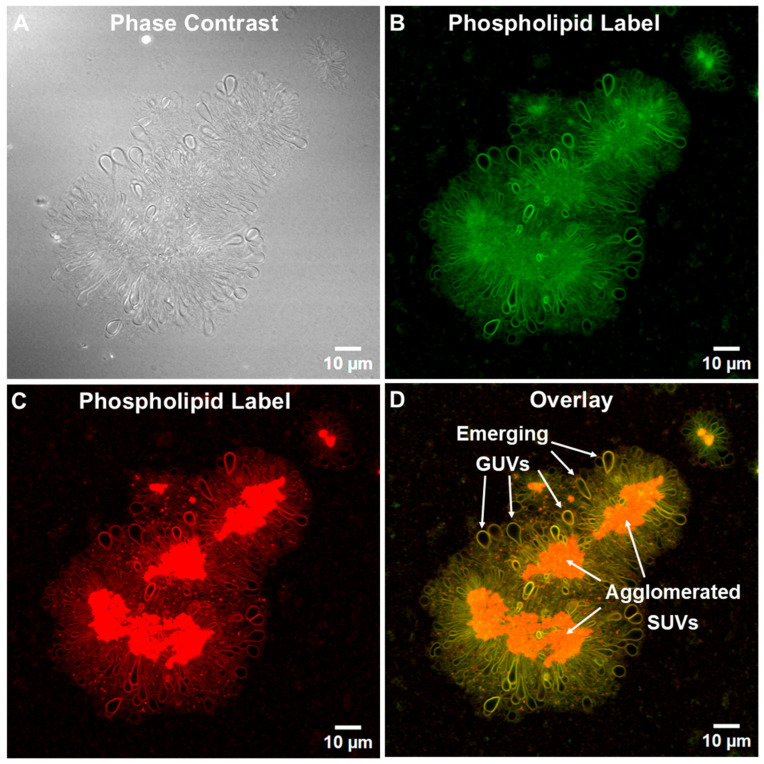
Visualization of TIMs in ALFQ by confocal microscopy. (**A**) Phase contrast image of TIMs. ALFQ labeled with 0.25 mol% Oregon Green™ 488 DHPE (**B**), 0.25 mol% Texas Red DHPE (**C**), and a merged image of the two channels with annotated structures (**D**). The scale bar is 10 μm, and the magnification is 60X.

Twenty-four hours later (Figure 4A), after the initial fusion had stopped and independent floating vesicles generated by the fusion reactions (including GUVs) had diffused away, well-defined stable phospholipid-labeled green (Figure 4B) or red (Figure 4C) images of TIMs were produced. When the images of the TIMs were overlaid, striking orange images revealed that the central core in each of the several TIM groupings contained phospholipids, but the individual SUVs in the central core were too small to be visualized (Figure 4D). The dense central areas of the TIMs were presumed to consist mainly of agglomerated SUVs and other small phospholipid fusion structures. Numerous round or tear-drop shaped structures were tethered to, and covered the surfaces of, the central cores, and these structures are presumed to represent emerging GUVs from the SUVs (Figure 4D).

**Figure 5:**
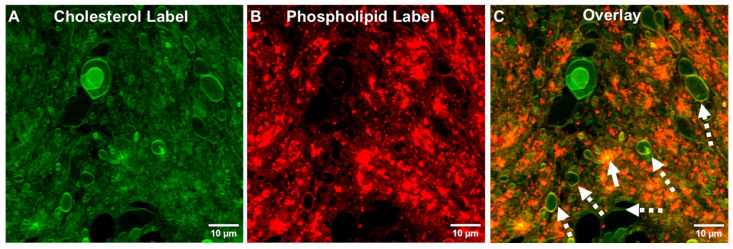
Visualization of phospholipids and cholesterol in TIMs of ALFQ by confocal microscopy. ALFQ cholesterol was visualized with TopFluor™ cholesterol (**A**), and with phospholipid using Texas Red™ DHPE (**B**), and a merged image is shown of the two channels (**C**). The solid arrow shows an example of a TIM structure; dashed arrows show free-floating GUVs. The vesicles show, on close examination, that they all contain both red phospholipid and green cholesterol chromophores.

As shown in the experiment in Figure 5, the initial ALF55 SUVs that had contained a green phospholipid-linked chromophore (Figure 4) were replaced by a green cholesterol-linked chromophore to form the green cholesterol-labeled SUVs. These cholesterol-labeled vesicles were then mixed with ALF55 SUVs containing a red phospholipid-linked chromophore. The resultant data in Figure 5 reveal that the membrane structures in ALFQ TIMs contain both cholesterol and phospholipid.

#### 3.1.3. Detection of TIMs Containing Both Phospholipids and Cholesterol in Lyophilized ALF55 in the Absence of QS21 Saponin

**Figure 6:**
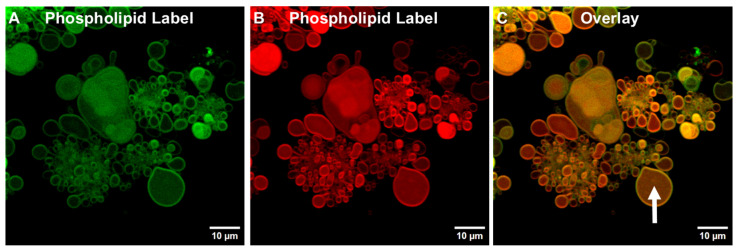
Visualization of phospholipids in TIMs and GUVs in lyophilized ALF55 (ALF55lyo) by confocal microscopy. ALF55lyo was labeled with green and red phospholipid chromophores, 0.25 mol% carboxyfluorescein-PE (**A**) and 0.25 mol% Cy5.5-PE (**B**), and a merged image is shown of the two channels (**C**). The arrow shows the cloudy interior of one, of most, tethered incomplete microspheres in TIM structures in ALF55lyo. These are all filled with phospholipid, which are likely phospholipid nanovesicles encapsulated during the growth of the giant spherules. Scale bar is 10 μm.

As noted in the Introduction, in a previous study, ALF55 SUV liposomes that had been lyophilized and then rehydrated could also generate free-floating GUVs (ALFlyo GUVs) [19]. Twenty-four hours after the initiation of fusion of lyophilized nanovesicles by hydration, and after rapid completion of the fusion process to form ALF55lyo, just as with ALFQ, stable TIMs remain (Figure 6). Figure 6 further reveals that upon labeling of the ALF55lyo TIMs, the stable membranes contained phospholipids. Upon overlaying the green (Figure 6A) and red phospholipid-stained images (Figure 6B) of ALF55lyo, just as with ALFQ TIMs, distinctive stable green- and red-labeled TIMs were observed, which were orange-colored after being overlaid (Figure 6C).

**Figure 7:**
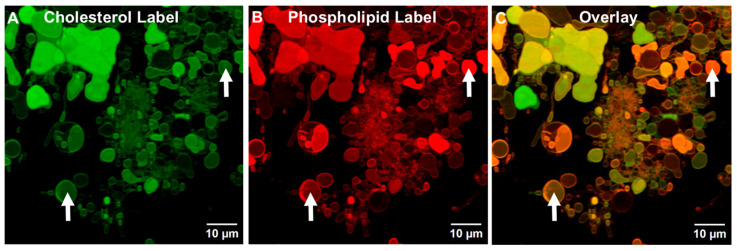
Visualization of TIMs in rehydrated lyophilized ALF55 (ALF55lyo) by confocal microscopy. ALF55lyo labeled with 0.25 mol% TopFluor^TM^ cholesterol (**A**), 0.25 mol% Cy5.5-PE (**B**), and a merged image of the two channels (**C**). Arrows indicate typical free-floating GUVs filled with both cholesterol (**A**) and phospholipid (**B**), which likely represent nanovesicles encapsulated in the GUVs. Scale bar is 10 μm.

In a further experiment, upon labeling the ALF55lyo TIM structures that contained a green cholesterol-linked chromophore (Figure 7A) and a red phospholipid chromophore (Figure 7B), and after overlaying the two images (Figure 7C), it was clear that the ALF55lyo TIM membranes, like ALFQ TIM membranes, contained both phospholipid and cholesterol.

However, unlike the apparently mainly empty tethered incomplete vesicles found in ALFQ TIMs (Figure 4D), the interior spaces of most (but not all) of the tethered vesicles in rehydrated ALF55lyo TIMs were homogeneously stained with both cholesterol and phospholipid chromophores, even though no individual smaller vesicles were visible in the interiors (see arrows in Figure 6 and Figure 7). Our interpretation of this is that during rehydration of lyophilized ALF55 particles leading to ALF55lyo GUVs and TIMs, some of the adjacent nanoliposomes may have been swept up and encapsulated as nanovesicles inside the emerging ALFlyo GUVs. That this was seen both in free-floating GUVs (Figure 6) and in TIMs (Figure 7) suggests further evidence that the GUVs had budded from the TIMs, rather than having been simply parallel independent structures.

#### 3.1.4. Cholesterol Is Not Required for Generating TIMs and GUVs in ALFlyo

**Figure 8:**
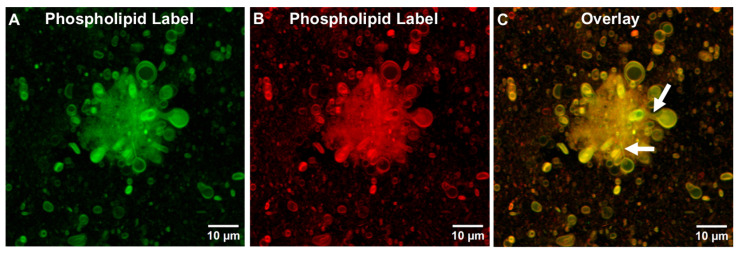
Visualization of TIMs in rehydrated lyophilized ALF0 (ALF0lyo) by confocal microscopy. ALF0lyo labeled with 0.25 mol% carboxyfluorescein-PE (**A**), 0.25 mol% Cy5.5-PE (**B**), and a merged image of the two channels (**C**). Arrows point out tubular nonlamellar stems often found during the fusion and budding process. Scale bar is 10 μm.

As shown in Figure 8, rehydrated ALFlyo liposomes that lacked cholesterol (ALF0lyo), but which contained one of two different phospholipid-bound chromophores (Figure 8A,B), revealed the presence of well-defined TIMs and GUVs, the shapes of which were similar when overlaid (Figure 8C). From this observation, we concluded that the presence of bulk phospholipid and MPLA in liposomes was sufficient after lyophilization, without the presence of cholesterol, for generation of TIMS and GUVs.

#### 3.1.5. Differential Centrifugation of ALF55lyo

As revealed in Table 1, relatively similar recoveries of total phospholipids were present in the pellet (50.5%) and the supernatant (54.6%). In contrast, both cholesterol and MPLA were recovered mainly in the pellets (cholesterol 54.3%; MPLA 71.7%) when compared to the supernatant (cholesterol 12.3%; MPLA 12.7%). These data thus indicated that both the cholesterol and MPLA migrated independently of the phospholipid into the emerging GUVs and TIMs during the fusion process of ALF55lyo.

Centrifugation of ALF55lyo at 8000 RPM (787× *g*) separated the GUVs + TIMs from the SUVs. The relative concentrations of total phospholipids, cholesterol, and MPLA were then examined in the supernatant and pellet by mass spectroscopy. The percent recovery was calculated relative to the original uncentrifuged ALF55lyo.

### 3.2. Comparative Influence of Cholesterol During the Generation of TIMs and GUVs in ALFQ and ALFlyo

**Figure 9:**
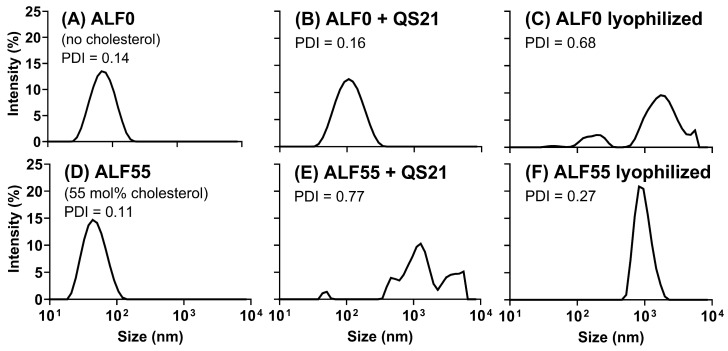
Influence of cholesterol on liposomal size as measured by dynamic light scattering. Size distribution graph for ALF lacking cholesterol (ALF0) (**A**) and ALF0 after the addition of QS21 (**B**) or after lyophilization and rehydration to obtain ALF0lyo (**C**). Size distribution graph for ALF55 (**D**) and ALF55 + QS21 after the addition of QS21 to form ALFQ (**E**) or after lyophilization and rehydration of ALF55 to obtain ALF55lyo (**F**). Polydispersity index (PDI) is shown for each formulation.

As shown in Figure 9A, in the absence of QS21, when spherical nanoliposomes containing MPLA, but lacking cholesterol (ALF0), were exposed to QS21 saponin (Figure 9B), only a slight broadening of the resultant median vesicle diameter distribution curve was observed, thus indicating that no GUVs were formed by QS21 in the absence of cholesterol. In contrast, when nanoliposomes containing MPLA and also containing 55 mol% cholesterol (ALF55) (Figure 9D) were exposed to QS21 (Figure 9E), it resulted in a polydisperse, increased wide diameter distribution that is typical for ALFQ.

In the absence of QS21, when ALF0 was lyophilized and then rehydrated (Figure 9C), a wide polydisperse population resulted that was similar to that in Figure 9E. However, when ALF55 (containing 55% cholesterol) was lyophilized and rehydrated in the absence of QS21 (Figure 9F), a narrow peak resulted, thus indicating a large number of particles with a median diameter of ~1000 nm.

We conclude that the similar results shown in Figure 9C (ALF0lyo) and Figure 9E (ALFQ) were due to mixtures of different sizes of TIMs together with free-floating vesicles having broad size distributions of large unilamellar vesicles (LUVs) (100−1000 nm), GUVs (≥1000 nm), and an irregular particle polydisperse size distribution, including nano, micro, and mega (>30,000 nm) size particles, likely including a wide range of sizes of spherical vesicles and TIMs.

### 3.3. Comparative Zeta Potentials of Liposomal Particles

Analysis of the zeta potentials in the various particles described in this study (Table 2) revealed that ALF55 and ALFQ both have similar negative zeta potentials, while the lack of cholesterol in ALF0 and ALF0 with QS21 contributed to slightly more negative values than were observed in the formulations with cholesterol. Upon lyophilization, however, both ALF0lyo and ALF55lyo exhibited comparable zeta potentials. When ALF55lyo was centrifuged at 787× *g* to obtain the supernatant and pellet fractions containing purified SUVs, as well as mixtures of GUVs and TIMs, respectively, a more negative zeta potential was observed in the pellet compared to the supernatant. As shown in Table 1, the ALF55lyo pellet contains most of the MPLA in the liposomes, which result in a more negative zeta potential.

## 4. Discussion

The surprising discovery that the self-assembly of ALFQ results in the production of GUVs was an important factor leading to the differentiation of ALFQ from other vaccine adjuvants and lipid structures containing phospholipids, cholesterol, and a saponin constituent. AS01, an adjuvant system created by GSK, like ALFQ, comprises liposomes containing both QS21 saponin and monophosphoryl lipid A as adjuvants [13]. However, ALFQ was assigned national and international patents at least in part because of its formation of GUVs during manufacture, as a unique difference from the SUVs of adjuvant system AS01 [22].

Here, a further discovery about ALFQ reveals that the self-assembly of GUVs is a multi-step process that occurs almost immediately after the addition of a micellar suspension of QS21 to an aqueous suspension of nano-size ALF55 liposomes (Video S1). We have discovered, in the course of the self-assembly of GUVs, the rapid assembly of a novel new intermediate structure, which is referred to here as a TIM. The entire production of both TIMs and GUVs occurs within seconds or minutes, but both the TIM and the GUV structures are stable. The apparent stability of the TIMs is likely due to the depletion of either or both of the MPLA and QS21 nonlamellar lipid constituents of the TIMs, including QS21/cholesterol structures, thus arresting the TIMs at the moment of nonlamellar lipid depletion.

Although the nanoscale events in the creation of ALFQ are not visible due to the diffraction limit of light microscopy, when seen in a time-lapse (Video S1), it is clear that within seconds after the addition of QS21 to the transparent suspension of nanoALF55 particles, intense cloudiness suddenly occurs. We conclude this cloudiness to be caused by the early-stage rapid nano-agglomeration of masses of individually invisible SUV particles. Future electron microscopy might help clarify these agglomerated nanostructures. Also shown in the supplemental video, visible GUVs and TIMs both rapidly emerged in view within seconds. Because both the GUVs and TIMs are microscale structures that are stable over a long period of time, large TIMs were easily visible after 6 h (Figure 3). Examination of multi-year-old previous preparations of ALFQ manufactured by a proprietary process, which was partly focused on the initial reduction and ultimate deconstruction of TIMs, succeeded in eliminating most, but not all, of the TIMs. In previous studies of ALFQ manufacturing by us using these proprietary methods under current Good Manufacturing Practices for human use, no TIMs were visible by light microscopy, thus demonstrating that TIMs can be degraded and removed from ALFQ, if necessary. However, further preliminary analysis of all the particles in a sample from a two-year old research preparation of ALFQ using our proprietary methods (not intended for human use), with a FlowCam^®^ instrument (as a demonstration, courtesy of Yokogawa Fluid Imaging Technologies, Scarborough, Maine, U.S.A.), showed the presence of a very small number of TIMs. These experiments suggest that TIMs are long-lived, but metastable structures. Further detailed analyses of the long-term stability of TIMs in ALFQ and ALFlyo and the implications for vaccine adjuvant activity, if any, will be undertaken in future studies.

It is clear that generation of TIMs and GUVs from nanoliposomes requires fusion of lipid bilayer membranes; it seems reasonable to believe that considerable insights about such fusion induced by nonlamellar lipids in a lamellar phospholipid environment can be provided by teachings of the field of lipid polymorphism [23,24,25,26,27,28]. Brandenburg and colleagues have pioneered studies on nonlamellar states of lipid A in water [29,30]. As summarized by Santos et al., citing research by Brandenburg et al., “Lipid-A can form different aggregation states in water, e.g., micellar (M), lamellar (L), hexagonal (H_I_), inverted hexagonal (H_II_), and nonlamellar cubic (Q), often with the co-existence of two states (e.g., Q and M)” [31].

In order to identify hypothetical mechanisms of self-assembly of ALF55 and ALFQ, it is useful to compare the sequential steps involved in each of their productions. First, in the creation of ALF55, the abovementioned aggregation states of MPLA in water theoretically might be created when the dried mixture of lipid constituents of ALF55 (containing MPLA) is initially hydrated to form an aqueous liposomal suspension. Second, as shown in the flowchart in Figure 2, a further sudden hydration method, so-called “forced hydration” [32], of the vesicles by microfluidization would involve high shear forces, high-pressure collisions of vesicles with each other and with the walls of the interaction chamber, vesicle distortion and fracturing of large lipid bilayers to form small ones, and intermixing of membranes with those of adjacent vesicles [32,33]. These sequential hydration episodes could perhaps allow creation of hydrated nonlamellar MPLA structures to occur both in the inner and outer leaflets of the liposomal bilayer, and perhaps even as nonlamellar structures in the center of the bilayer.

Upon initial hydration, ALF55 contains 55 mol% cholesterol when compared to total phospholipid. However, the hydrated DMPC-containing nanoliposomal lipid bilayer reportedly has more cholesterol in the inner leaflet than the outer leaflet [34]. The initial event in the self-assembly of TIMs and GUVs after adding QS21 saponin to lamellar nanoliposomes is that the QS21 saponin binds to the cholesterol molecules on the outer leaflet of the nanoliposome bilayer, thus replacing cholesterol with a new type of nonlamellar cholesterol/QS21 saponin structure. This might interfere with the interleaflet coupling of free cholesterol, which theoretically might trigger increased flip/flop of free cholesterol from the inner leaflet to the outer leaflet of the bilayer [35]. During the hypothetical fusion process, this might be a partial explanation for the ultimate movement of free cholesterol from SUVs to GUVs.

Our present data, which were obtained by confocal and fluorescent microscopic visualization of microdomains (Figure 3, Figure 4, Figure 5, Figure 6, Figure 7 and Figure 8), together with our previous discovery that the distribution of most of the total cholesterol, QS21 saponin, and MPLA was directed away from nanosized phospholipid liposomes into microsized phospholipid liposomes [2], provide a hypothetical sequence of nanodomain events fueled by the appearance of nonlamellar lipids in TIM and GUV structures to be considered. Some of the initial and sequential steps that might occur are as follows.

The generation of GUVs involved TIMs that initially served as a transitional step, but which were ultimately left behind as long-lived mega-size structures (some > 200 µm) containing aggregated incomplete vesicular structures tethered together in the form of metastable TIMs. The mega-size TIM structures contain multiple reiterations of giant tear-drop and spherical closed-membrane structures loaded with pathogen-associated molecular patterns (PAMPs) or danger- or damage-associated molecular patterns (DAMPs) in the form of MPLA and QS21 embedded in aggregated groups of giant liposomes composed of phospholipids with saturated fatty acyl groups. The nonlamellar sites of the liposomal lamellar membranes, together with the mega-size (> 30,000 nm) aggregations of the TIMs themselves, could be reasonably considered to be a form of danger or damage that might initiate the activation of innate immunity.

Theoretical mechanisms of the vesiculation and budding of lipid membranes have proposed that cholesterol-bound hydrophilic sugars of saponins interact strongly together to cause tightly aggregated clumps [26,27,36]. It was further proposed by these authors that with lipid membranes containing saponins, vesiculation occurs only at the outer leaflet of membrane sites that contain aggregated saponin molecules bound to cholesterol. These ideas seem plausible. In view of this it seems reasonable to suggest that the visible outer leaflets of the bilayers of giant vesicular membranes in ALFQ might contain most, or virtually all, of the cholesterol and QS21, of the micro- and mega-size GUVs and TIM structures that are shown here visually. However, it also seems plausible to suggest that during the original hydration of dried lipids to form ALF55, and the subsequent reduction of large vesicles to small ones during highly stressful conditions caused by microfluidization of ALF55, the nonlamellar forms of MPLA might be present both on the inner and outer leaflets of the bilayers of both ALFQ and ALFlyo.

Lyophilization of ALF55 SUVs caused the generation of GUVs and TIMs that were seemingly similar to those formed by simply adding QS21 to ALF55 to form ALFQ (compare Figure 4 with Figure 6; and Figure 9C with Figure 9E). However, the underlying mechanisms of forming these ALFQ and ALFlyo structures were different in at least two ways. First, ALFQ requires the presence of cholesterol to form GUVs (see ALF0 in Figure 9B vs. ALF55 in Figure 9E), but ALF55lyo does not require cholesterol to generate GUVs (see ALF0lyo in Figure 8 and Figure 9C). Second, the presence of cholesterol dramatically narrowed the polydisperse distribution of visible particles in ALF55lyo to a median diameter of ~1000 nm (see ALF0lyo in Figure 9C vs. ALF55lyo in Figure 9F).

Through the detailed studies of Gregory Gregoriadis and colleagues, it has long been known that lyophilization and rehydration of pre-formed GUV liposomes can result in giant liposomes with huge encapsulation capacities [37,38,39,40]. When pre-formed GUVs, with a mean diameter of 5.5 µm, were mixed with particles as large as bacterial cells, all of which were dehydrated and rehydrated, the large particles (such as bacteria) were encapsulated within the giant liposomes [38]. However, in contrast to other liposomal lyophilization methods that start with large vesicles, as shown in Figure 2, in the present study, the self-assembly of ALF55lyo started only with ALF55 (containing MPLA) that was first microfluidized to form SUVs, and the SUVs were then lyophilized and rehydrated to form TIMs containing aggregated incomplete vesicles; free-floating GUVs then budded from the incomplete vesicles. We conclude that the presence of nonlamellar MPLA in ALF55 SUVs, which were then lyophilized and rehydrated (ALF55lyo), initiated the agglomeration and fusion of SUVs to generate GUVs that budded from the intermediary TIM structures.

A further conclusion regarding why stable TIMs occurred is that, upon depletion of critical non-lamellar lipids, such as QS21 or hydrated MPLA (in ALFQ), or hydrated MPLA alone (in ALF55lyo), the intermediary fusion process that was occurring in the TIMs stopped, and this left metastable incomplete TIMs as visual evidence of the underlying nanoscale fusion processes that generated GUVs from SUVs in both ALFQ and ALF55lyo.

## Figures and Tables

**Table 1:** Recovery by weight (mg) of total phospholipid, cholesterol, and MPLA after centrifugation of ALF55lyo.

Component	Uncentrifuged ALF55lyo (mg)	Centrifuged ALF55lyo			
		Pellet (mg)	% Recovery	Supernatant (mg)	% Recovery
Total Phospholipid	75.53 ± 0.57	38.15 ± 1.18	50.5	41.25 ± 1.35	54.6
Cholesterol	48.22 ± 3.66	26.21 ± 0.6	54.3	5.94 ± 0.07	12.3
MPLA	2.15 ± 0.08	1.54 ± 0.02	71.7	0.27 ± 0.00	12.7

**Table 2:** Zeta potentials of liposome formulations.

Liposome	Zeta Potential (mV)
ALF55	−11.13 ± 0.76
ALFQ	−12.73 ± 0.21
ALF0	−16.20 ± 1.51
ALF0 + QS21	−14.37 ± 0.84
ALF0lyo	−15.03 ± 0.60
ALF55lyo	−14.97 ± 0.96
ALF55lyo pellet ^1^	−14.20 ± 0.26
ALF55lyo supernatant ^1^	−12.40 ± 0.69

^1^ After centrifugation of ALF55lyo at 787× *g* as shown in Table 1.

## Data Availability

All data are available from the authors.

## Supplementary Materials

The following supporting information can be downloaded at: https://www.mdpi.com/article/10.3390/pharmaceutics17091092/s1, Video S1: Video showing the self-assembly of GUVs and TIMs following QS21 addition.
