# Supplementary material for: Mechanisms of Self-Assembly of Giant Unilamellar Vesicles in the Army Liposome Formulation (ALF) Family of Vaccine Adjuvants
Source: Pharmaceutics. 2025 Aug 22;17(9):1092. doi: 10.3390/pharmaceutics17091092 (PMC12473096; doi:10.3390/pharmaceutics17091092)
Supplement: Supplementary file 1 [file pharmaceutics-17-01092-s001.zip › pharmaceutics-3765766-SI-proofreading.pdf]

pharmaceutics

Article

Mechanisms of Self-Assembly of Giant Unilamellar Vesicles in the Army Liposome Formulation (ALF) Family of Vaccine Adjuvants

Calin Nicolescu [1,2]*, Essie Komla [1,2], Mangala Rao [1], Gary R. Matyas [1] and Carl R. Alving [1]*

[1] U.S. Military HIV Research Program, Center for Infectious Disease Research, Walter Reed Army Institute of Research, 503 Robert Grant Avenue, Silver Spring, MD 20910, United States; ekomla@hivresearch.org (E.K.); mrao@hivresearch.org (M.R.); gmatyas@hivresearch.org (G.R.M.)

[2] Henry M. Jackson Foundation for the Advancement of Military Medicine, 6720A Rockledge Drive, Bethesda, MD 20817, United States

* Correspondence: Correspondence: calving@hivresearch.org (C.R.A.), cnicolescu@hivresearch.org (C.N.)

Supplementary Video

Video S1: Video showing the self-assembly of GUVs and TIMs following QS21 addition. The video was taken over the course of five minutes and recorded as a 1.5x timelapse directly after QS21 was added to ALF55. Aggregation of SUVs was observed, with TIMs and GUVs becoming visible shortly thereafter. Magnification was 40X, and focus was not adjusted throughout the video.

Academic Editors: Juliana De Oliveira Silva, Caroline Mari Ramos Oda, Elaine Amaral Leite

Received: 2 July 2025
Revised: 6 August 2025
Accepted: 18 August 2025
Published: 22 August 2025

Citation: Nicolescu, C.; Komla, E.; Rao, M.; Matyas, G.R.; Alving, C.R. Mechanisms of Self-Assembly of Giant Unilamellar Vesicles in the Army Liposome Formulation (ALF) Family of Vaccine Adjuvants. *Pharmaceutics* **2025**, *17*, 1092. https://doi.org/10.3390/pharmaceutics17091092

Copyright: © 2025 by the authors. Licensee MDPI, Basel, Switzerland. This article is an open access article distributed under the terms and conditions of the Creative Commons Attribution (CC BY) license (https://creativecommons.org/licenses/by/4.0/).
